# Urinary Gamma-Glutamil Transferase as an Early Biomarker of Renal Disease in Dogs with Leishmaniosis

**DOI:** 10.3390/vetsci12050436

**Published:** 2025-05-02

**Authors:** Patricia Ruiz, Inmaculada Sevidane, Angela Durán, Ana Belén García, Beatriz Macías-García, Rafael Barrera

**Affiliations:** 1 MINVET Research Group, Departamento de Medicina Animal, Facultad de Veterinaria, Universidad de Extremadura, 10003 Cáceres, Spain; patriciart@unex.es (P.R.); rabacha@unex.es (R.B.); 2 Hospital Clínico Veterinario, Facultad de Veterinaria, Universidad de Extremadura, 10003 Cáceres, Spain; inmasevidane97@gmail.com (I.S.); angeladg@hotmail.es (A.D.); anabelengarcia123@gmail.com (A.B.G.)

**Keywords:** canine leishmaniosis, chronic kidney disease, early biomarker, uGGT/c ratio

## Abstract

Canine leishmaniosis, caused by *Leishmania infantum*, is a zoonotic disease often leading to chronic kidney disease (CKD). Early detection of renal damage is critical for prognosis and treatment, as conventional biomarkers, such as serum creatinine and symmetric dimethylarginine (SDMA), are inadequate for detecting early stages of renal dysfunction. This study evaluates the urinary gamma-glutamyl transferase-to-creatinine ratio (uGGT/c) as an early biomarker for renal injury in dogs with leishmaniosis, including 17 healthy dogs and 77 dogs with leishmaniosis, categorized using the International Renal Interest Society (IRIS) guidelines. Plasma and urinary parameters, including uGGT/c, were analyzed. Elevated uGGT/c ratios were observed in over 50% of non-azotemic and non-proteinuric dogs and all dogs with mild-to-moderate proteinuria. The uGGT/c ratio demonstrated a 90.4% sensitivity and 80% specificity for early detection of renal tubular damage, outperforming plasma creatinine (44.2%) and SDMA (51.9%). The uGGT/c ratio correlated strongly with proteinuria (UPC; r = 0.716, *p* < 0.001) and SDMA (r = 0.591, *p* < 0.001), indicating its usefulness as an early indicator of renal dysfunction. Despite the small sample size, the implementation of the uGGT/c ratio as promising biomarker could improve early diagnosis and clinical intervention, helping to avoid irreversible renal damage in dogs affected by leishmaniosis.

## Simple Summary

Canine leishmaniosis (CanL), caused by *Leishmania infantum*, is a zoonotic disease often associated with chronic kidney disease (CKD) due to immune complex deposition leading to glomerular and tubular injury. Early detection of renal damage is crucial, as conventional biomarkers like serum creatinine and symmetric dimethylarginine (SDMA) lack sensitivity in early CKD stages. This study evaluates the urinary gamma-glutamyl transferase-to-creatinine ratio (uGGT/c) as an early biomarker of renal tubular damage in 94 dogs (17 healthy controls and 77 naturally infected). Dogs were classified using the International Renal Interest Society (IRIS) CKD staging guidelines, and plasma and urinary parameters were analyzed. Elevated uGGT/c ratios were detected in over 50% of non-azotemic, non-proteinuric dogs (IRIS 1_NP_) and all dogs with mild-to-moderate proteinuria (IRIS 1_P_). The uGGT/c ratio demonstrated 90.4% sensitivity and 80% specificity, outperforming creatinine (44.2%) and SDMA (51.9%). Strong correlations were observed between uGGT/c and proteinuria (r = 0.716, *p* < 0.001) and uGGT/c and SDMA (r = 0.591, *p* < 0.001). Despite the small sample size, the inclusion of uGGT/c ratio in diagnostic protocols could improve early CKD detection and intervention, preventing irreversible damage.

## 1. Introduction

Canine leishmaniosis (CanL) is a zoonotic disease caused by the protozoan parasite Leishmania infantum, which is endemic to Spain [1] and transmitted by female phlebotomine sandflies. Affected animals exhibit a wide spectrum of clinical manifestations ranging from subclinical infection with no apparent signs to severe disease, which can be life-threatening. Chronic renal disease (CKD) is an irreversible and progressive condition characterized by the loss of secretory and excretory nephron functions, thereby compromising overall renal functionality [2], which is one of the most severe complications and is the mean cause of mortality in dogs with CanL [3]. Renal failure in CanL is associated with the deposition of soluble circulating immune complexes, leading to glomerular disease [4]. Clinically, this condition presents as persistent proteinuria, often progressing to glomerulonephritis and/or interstitial nephritis [5] and ultimately culminating in CKD.

The diagnosis of renal disease in canine medicine typically relies on standardized blood and urine biomarkers, such as serum creatinine, symmetric dimethylarginine (SDMA), urine specific gravity, and the urine protein-to-creatinine ratio (UPC). These markers are recommended by the International Renal Interest Society (IRIS) for the evaluation and staging of renal damage (https://www.iris-kidney.com/iris-guidelines-1) (accessed on 20 April 2025) and have been widely used in the clinical classification of CanL [6]. However, these biomarkers are usually altered only when renal disease has progressed to advanced stages, at which point the condition may already be irreversible. SDMA has been proposed as a more sensitive marker due to its lower susceptibility to non-renal factors and its ability to detect early decreases in glomerular filtration rate (GFR) [7,8]. Nevertheless, there is an urgent need for novel biomarkers capable of detecting kidney damage at earlier stages. Such markers should ideally reflect reduced GFR through changes in specific serum proteins or the presence of specific urinary enzymes.

Among promising serum biomarkers, cystatin C [9,10,11] and neutrophil gelatinase-associated lipocalin (NGAL) [12,13,14] have been studied for their potential to detect early renal dysfunction. Enzymatic markers in urine, such as N-acetyl-beta-D-glucosaminidase (NAG) [11,15] and gamma-glutamyl transferase (GGT) [15,16,17], have also shown a promising potential for early renal disfunction diagnosis. GGT has been extensively studied in dogs with acute kidney injury (AKI) and is associated with tubular damage [18]. GGT is localized at the brush border of proximal tubular epithelial cells, and increases in urinary GGT concentrations are attributed to tubular epithelial cell injury [19]. However, this is only applicable when the glomerular barrier is intact, as glomerular disease may result in serum protein leaking into the ultrafiltrate, including GGT [20].

The enzymatic activity of GGT in urine remains stable under specific conditions, with acceptable variability (inter-assay coefficient of variation <7.74%) when stored at 20 °C for 3 days, 4 °C for 5 days, and −80 °C for up to 14 days. Furthermore, GGT activity is not significantly affected by the presence of hemoglobin, whole blood contamination, or microbial contamination (e.g., *Escherichia coli* or *Enterococcus* sp.) [20]. For accurate interpretation, GGT activity is typically expressed as a ratio to urinary creatinine (uGGT/c), allowing adjustment for urinary flow variations [21]. Urine pH also influences GGT stability, with enzymatic activity decreasing at pH < 5 or >8.5, optimal stability observed between pH 6.5 and 8.0 [20,22], and with significant differences in urinary GGT concentrations reported between acidic and basic samples [23].

To date, only three studies have evaluated the usefulness of the uGGT/c ratio in dogs with *L. infantum* infection. Ibba et al. (2016) [16] have reported a significant increase in uGGT/c ratio in dogs exhibiting tubular-origin proteinuria, with values sevenfold higher than those observed in cases of glomerular proteinuria, highlighting its potential as a tubular damage marker. Furthermore, de Oliveira Frazilio et al. (2018) [24] have found that both urinary GGT (uGGT) and the uGGT/c ratio were more sensitive than traditional analyses for detecting renal damage in dogs with visceral leishmaniosis. However, some studies have focused on the uGGT/c ratio as a monitoring tool rather than an early diagnostic marker. For instance, Pardo-Marín et al. (2017) [15] observed no significant reduction in the uGGT/c ratio following treatment with N-methylglucamine antimoniate and allopurinol. Paltrinieri et al. (2018) [17] demonstrated that persistent elevations in uGGT/c following treatment indicate ongoing tubulointerstitial damage. Despite previous studies investigating the use of urinary GGT as a biomarker for kidney disease in dogs affected by Leishmaniosis, our study focuses on its potential for early detection in the initial stages of chronic kidney disease (CKD). In daily clinical practice, more accurate biomarkers are needed for the early detection and location of kidney damage within the nephron, particularly in dogs with leishmaniosis that do not yet exhibit clinical or analytical signs of renal damage. Therefore, the primary objective of this study was to verify the usefulness of uGGT/c ratio in kidney disease diagnosis at the earlier stages of leishmaniosis, specifically in animals that are asymptomatic, exhibit normal clinicopathological parameters, and have not received previous treatment for CanL. Our aim was to evaluate whether this biomarker could identify renal dysfunction in dogs that would otherwise be missed by conventional methods.

## 2. Materials and Methods

### 2.1. Animals

A cross-sectional clinical study was conducted at the University of Extremadura, Spain, between January 2022 and April 2024. Written informed consent was obtained from all dog owners, as required. Client-owned dogs of any breed and age with a previous diagnosis of leishmaniosis were included in the study. These dogs were either referred or presented as primary patients at the Veterinary Clinical Hospital (VCH) of the University of Extremadura. Ethical approval for the study was deemed unnecessary by the Animal Experimentation Ethics Committee, as the Bioethics Committee determined that no experimental procedures were involved (Internal Record Number 91/2020), in accordance with RD 53 of 2013, because the blood and urine samples used were collected from clinical patients admitted to the hospital and were required for routine diagnostic purposes.

A total of ninety-four dogs were included in the study, which were divided into two groups: a control group consisting of 17 clinically healthy dogs, and a diseased group comprising 77 naturally infected dogs diagnosed with *Leishmania infantum*. The control group included dogs presented for gonadectomy or routine annual check-ups requested by the tutor, all of which tested negative for *L. infantum*. The diseased group comprised dogs that met the following inclusion criteria: (i) serologically positive for leishmaniosis, (ii) aged over 12 months, and (iii) with no concurrent diseases. A total of 110 dogs were initially evaluated, from which 33 were excluded due to the presence of concurrent diseases (*n* = 14), urinary pH values outside the refence range (5.5–8.5) (*n* = 2), previous treatment with leishmanicidal or leishmaniostatic drugs (*n* = 10), active urinary tract infection (positive urine culture) (*n* = 4), and clinical signs of dehydration (*n* = 3).

The selection of dogs was randomized through a simple random sampling method, ensuring that all eligible dogs were equally likely to be included in the study. The dogs were randomly assigned to either the control or diseased group based on their health status and *Leishmania* diagnosis, without any prior bias or stratification. All patients were recruited consecutively and none declined participation. No losses occurred during the inclusion phase. The dogs with urinary pH values lower than 5.5 or higher than 8.5 were excluded from the analysis due to potential enzyme instability [20,22], and all dogs in both groups exhibited normal hydration status, no urinary tract infections, and had never been treated with leishmanicidal or leishmaniostatic drugs, if they did not meet requirements, as mentioned above, they were excluded from the study. Each dog underwent a thorough clinical evaluation, including physical examination, measurement of systolic arterial blood pressure, complete blood count, serum biochemical analysis, urinalysis (including urine culture), and fecal examination for parasites.

The classification of the dogs included in the study was performed according to the International Renal Interest Society (IRIS) guidelines (https://www.iris-kidney.com/iris-guidelines-1) (accessed 20 April 2025), based on plasma creatinine concentrations and adjusted using blood levels of symmetric dimethylarginine (SDMA). According to IRIS guidelines for chronic kidney diseases (CKD), Stage 1 is defined by a creatinine concentration of <1.4 mg/dL and an SDMA concentration <18 µg/dL, or the presence of other renal abnormalities (proteinuria or abnormal renal imaging findings), despite normal creatinine. Stage 2 is defined by a creatinine concentration of 1.4–2.8 mg/dL, with typical SDMA values of 18–35 µg/dL. Stage 3 includes creatinine concentrations of 2.9–5.0 mg/dL and SDMA values of 36–54 µg/dL. Stage 4 is defined by creatinine concentrations >5.0 mg/dL and SDMA >54 µg/dL. Each IRIS stage was further sub-staged, as follows, based on the presence or absence of proteinuria using the urinary protein-to-creatinine (UPC) ratio: non-proteinuric (UPC < 0.2), borderline proteinuric (UPC 0.2–0.5), and proteinuric (UPC > 0.5). This sub-staging helps refine prognosis and therapeutic decisions by accounting for the impact of proteinuria on disease progression.

### 2.2. Clinical Pathology Testing

Blood samples were collected following the hospital’s standard protocol, either from the jugular or cephalic vein, depending on the case, after a 12 h fasting period. Samples were drawn into EDTA-K3 tubes (for hematological analysis) and heparinized tubes (for plasma biochemistry). The latter were centrifuged at 540× *g* for 10 min to obtain plasma. Hematological analyses were performed immediately using an automated analyzer (ProCyte DX^®^; IDEXX Laboratories, Westbrook, ME, USA) and were complemented by blood smears stained with Diff-Quick. The parameters assessed included red blood cell count, hemoglobin concentration, hematocrit, mean corpuscular volume (MCV), mean corpuscular hemoglobin concentration (MCHC), total leukocyte count, differential leukocyte count (neutrophils, eosinophils, basophils, lymphocytes, monocytes), and platelet count.

Biochemical variables measured included urea, creatinine (pCreat), alanine aminotransferase (ALT), SDMA, cholesterol, phosphorus, total protein, and albumin. Globulin concentration was calculated by subtracting the albumin value from the total protein value. These determinations were performed using commercial kits (Spinreact^®^, Barcelona, Spain) and an automated biochemical analyzer (Spin200E, Spinreact^®^, Barcelona, Spain), following the manufacturer’s instructions. SDMA was analyzed using a Catalyst Dx Chemistry Analyzer (IDEXX^®^ Laboratories, Inc., Westbrook, ME, USA).

Urine samples were obtained via ultrasound-guided cystocentesis. A 0.5 mL aliquot was used for urine culture, and 3 mL were employed for routine urinalysis (Multistix Reagent Strips^®^, Bayer Corporation, Madrid, Spain) according to the manufacturer’s instructions, using an Urispin reader (Spinreact^®^, Barcelona, Spain). The remaining urine was centrifuged at 130× *g* for 5 min to obtain the urinary sediment, which was examined under an optical microscope (40× objective). The sediment was considered inactive if it met the following criteria: <5 erythrocytes/hpf, <5 leukocytes/hpf, occasional epithelial cells, and absence of bacteria. The urine specific gravity (USG) was determined using a refractometer (ZUZI 300). To calculate the urine protein-to-creatinine ratio (UPC), protein concentration was determined using the pyrogallol-molybdate red method, and creatinine concentration was measured using the Jaffé reaction (RAL Diagnostics^®^, SA, Barcelona, Spain), both performed on an automated analyzer (Spin200E; Spinreact^®^, Barcelona, Spain). Lastly, urinary GGT concentrations were determined using an automated blood chemistry analyzer (Spin200E, Spinreact^®^, Barcelona, Spain). The GGT determination principle involves catalyzing the transfer of the γ-glutamyl group from γ-glutamyl-p-nitroanilide to glycylglycine, with the rate of 2-nitro-5-aminobenzoic acid formation being proportional to the GGT concentration present in the sample. The results of urinary GGT were expressed as a ratio with creatinine: uGGT/c (U/g).

### 2.3. Diagnosis of Leishmaniosis

In all dogs included in the study, a semi-quantitative enzyme-linked immunosorbent assay (ELISA) was performed to detect antibodies against the total soluble antigen of *Leishmania infantum* (derived from *L. infantum* promastigotes, strain MCAN/ES/1996/BCN150, zymodeme MON-1) [25]. CanL was diagnosed based on the presence of compatible clinical signs and a serological antibody titer at least three times higher than the laboratory’s reference cutoff value. This diagnosis was further supported by the direct microscopic identification of *Leishmania* amastigotes in lymph node or bone marrow aspirates (3 out of 77 dogs; 3.9%) and by positive PCR results (15 out of 77 dogs; 19.5%). Additionally, all dogs were tested for the absence of canine heartworm disease, *Anaplasma phagocytophylum*, *Borrelia burgdorferi*, and *Ehrlichia canis* antibodies using a commercial ELISA method (Canine SNAP 4Dx test; IDEXX Laboratories, Westbrook, ME, USA).

### 2.4. Statistical Analyses

Statistical analyses were performed using SPSS Statistics software, version 27 (IBM Corp., Armonk, NY, USA). Data are presented as mean ± standard deviation or median with its corresponding interquartile range (IQR) to describe the distribution of continuous variables. The distribution of the assayed parameters was assessed using the Kolmogorov–Smirnov test. Comparisons between groups were performed using the Mann–Whitney U test, followed by Dunn’s post-hoc test.

The continuous variables compared between the control and diseased groups included hematological parameters (such as hemoglobin and hematocrit), biochemical parameters (urea, pCreat, cholesterol, calcium, phosphorus, total protein, SDMA, albumin, albumin-to-globulin ratio, globulins, ALT, ALKP, sodium, potassium, chlorine), and urinary parameters (USG, UPC, and uGGT/c). Categorical variables, such as the presence of proteinuria, were analyzed using the chi-square or Fisher’s exact test, as appropriate. Correlations between biomarkers and conventional parameters were calculated using Spearman’s rank correlation coefficient.

Receiver operating characteristic (ROC) curves were constructed to evaluate the predictive capacity of urinary GGT and other biomarkers, such as serum creatinine, SDMA and UPC, for the early detection of CKD. The optimal cut-off point for each biomarker was selected using the Youden index, which maximizes both sensitivity and specificity. Sensitivity and specificity were determined based on previously validated criteria [26] and were reported alongside their corresponding 95% confidence intervals (CIs) to indicate the precision of each biomarker in detecting early stage CKD in dogs with leishmaniosis. The area under the curve (AUC) and 95% confidence intervals (CIs) were also calculated. For the purpose of the ROC analysis, the control group was classified as healthy (negative) and the diseased group as Leishmania-infected dogs (positive), focusing on the early detection of renal damage in infected dogs, including those without azotemia or overt renal failure. Statistical significance was set at *p* < 0.05.

## 3. Results

Of the 94 dogs included in this study, 17 were healthy controls with a mean age of 5.12 years (range: 2–10 years). This group comprised 9 males (6 castrated, 3 intact) and 8 females (5 spayed, 3 intact). The remaining 77 dogs were diagnosed with leishmaniosis, with a mean age of 5.75 years (range: 2–11 years), consisting of 45 males (26 castrated, 19 intact) and 32 females (17 spayed, 15 intact). Based on the IRIS classification system (IRIS Guidelines), these dogs were categorized into the following stages: 28 dogs (36.36%) in IRIS stage 1; 19 dogs (24.68%) in IRIS stage 2; 13 dogs (16.88%) in IRIS stage 3; and 17 dogs (22.08%) in IRIS stage 4.

IRIS 1 stage was further subdivided into two subgroups based on the presence or absence of measurable proteinuria, as recommended by IRIS—non-proteinuric (IRIS 1_NP_; *n* = 10; UPC < 0.2) and proteinuric dogs (IRIS 1_P_; *n* = 18; UPC ≥ 0.2). Borderline proteinuric dogs (UPC: 0.2–0.5) and proteinuric dogs (UPC > 0.5) were included in the IRIS 1_P_ subgroup.

In the group of dogs with leishmaniosis, the most frequently observed clinical signs were lymphadenomegaly, weight loss, anorexia, exfoliative dermatitis and ulcers over bony prominences. On some occasions, some dogs also presented epistaxis, temporal muscle atrophy and uveitis, as well as polyuria, polydipsia and digestive signs in more advanced stages of the disease.

### 3.1. Hematology

The most representative hematological results are summarized in Supplementary Table S1. Control group values were within the normal reference ranges. In the leishmaniosis groups, there was a tendency toward normocytic, normochromic anemia, with statistically significant differences observed across all stages of the disease when compared with the control, as expected. A significant decrease in lymphocyte counts was noted in IRIS stage 4, and eosinophil counts decreased significantly in stages IRIS 1_P_, 2, 3, and 4.

### 3.2. Plasma Biochemical Parameters

Descriptive statistics for plasma parameters (mean ± SD) are presented in Table 1. Total protein levels showed a statistically significant increase in stage IRIS 1_P_ only, while globulin levels increased significantly in all stages except for IRIS 1_NP_. Conversely, albumin levels decreased significantly across all leishmaniosis stages when compared with controls, and the albumin-to-globulin ratio followed a similar trend, with the exception of IRIS 1_NP_. Creatinine, urea, and SDMA levels were significantly elevated in stages IRIS 2, 3, and 4.

**Figure 1:**
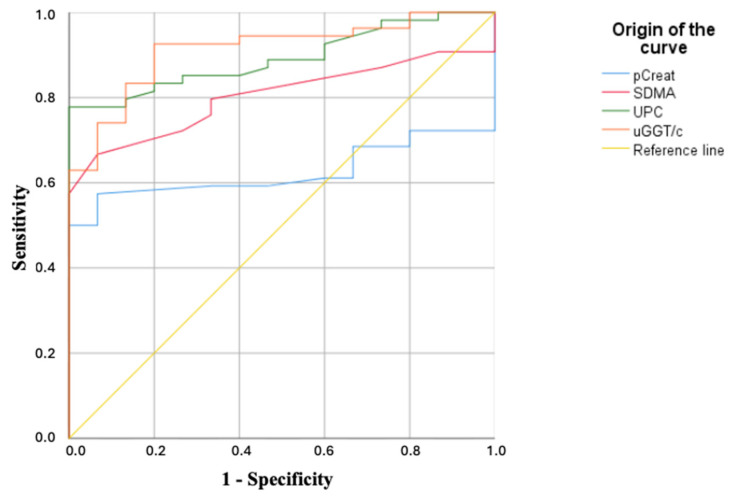
Nonparametric ROC curves comparing the diagnostic accuracy of pCrea, SDMA, UPC, and uGGT/c in detecting kidney impairment in all dogs affected by CanL. Abbreviations: pCreat, plasmatic creatinine; SDMA, symmetric dimethylarginine; UPC, urinary proteins/creatinine ratio; uGGT/c, urinary gamma-glutamyl transferase/creatinine ratio.

For pCreat, the median concentrations were 1.16 mg/dL (IQR: 1.00–1.20 mg/dL) in the control group, 0.96 mg/dL (IQR: 0.73–1.15 mg/dL) in the overall IRIS stage 1 group, 1.10 mg/dL (IQR: 0.98–1.25 mg/dL) in the IRIS 1_NP_ subgroup, and 0.90 mg/dL (IQR: 0.70–1.06 mg/dL) in the IRIS 1_P_ subgroup. The receiver operating characteristic (ROC) curve analysis established a cut-off value of 1.38 mg/dL (Table 2; Figure 1) and demonstrated that no dogs in the control group or in IRIS stage 1 exceeded the determined threshold for the total cohort. However, 13 out of 19 dogs (68.42%) in IRIS stage 2 and all dogs in stages 3 and 4 (100%) exhibited values above this cut-off.

The median plasma SDMA concentrations were 8 µg/dL (IQR: 7.75–11.0 µg/dL) in the control group, 9.00 µg/dL (IQR: 7.00–12.00 µg/dL) in the overall IRIS stage 1 group, 8.50 µg/dL (IQR: 5.50–11.00 µg/dL) in the IRIS 1_NP_ subgroup, and 10.00 µg/dL (range: 8.00–12.00 µg/dL) in the IRIS 1_P_ subgroup. ROC curve analysis established a cut-off value of 13.5 µg/dL (Table 2; Figure 1) and demonstrated that no dogs in the control group or in the IRIS 1_NP_ subgroup exceeded the threshold determined for the total cohort. However, 16 out of 18 dogs (88.89%) in the IRIS 1_P_ subgroup, 17 out of 19 dogs (89.48%) in the IRIS stage 2, and all dogs in stages 3 and 4 (100%) presented values above the calculated cut-off.

### 3.3. Urinary Parameters

Descriptive statistics for urinary parameters (mean ± SD) are presented in Table 3. Urine pH was found to influence urine GGT activity [23]. Among the 17 healthy controls, the mean uGGT/c index was 6.64 ± 3.40 U/g in dogs with urine pH < 7.0 (*n* = 6) and 13.38 ± 10.21 U/g in dogs with urine pH ≥ 7.0 (*n* = 11). This difference was not statistically significant (*p* = 0.085).

The UPC showed a significant increase starting from IRIS stage 1_P_, while the USG decreased significantly in IRIS stages 1_P_, 2, 3, and 4. The uGGT/c ratio also increased significantly in comparison to the control group in all groups.

For UPC, the median values were 0.12 (IQR: 0.07–0.20) for the control group, 0.49 (IQR: 0.13–1.87) for IRIS 1 (including all proteinuric and non-proteinuric patients), 0.11 (IQR: 0.10–0.15) for IRIS 1_NP_, and 1.69 (IQR: 0.64–3.84) for IRIS 1_P_. No dogs in the control group or IRIS 1_NP_ exceeded the cut-off value of 0.22 (Table 2). In contrast, all dogs in IRIS stages 1_P_, 2, 3, and 4 (100%) exceeded this threshold.

For the uGGT/c, the median levels were 9.54 U/g (IQR: 3.67–14.13 U/g) in the control group, 32.85 U/g (IQR: 22.14–55.30 U/g) in the overall IRIS stage 1 group, 22.15 U/g (IQR: 7.34–35.52 U/g) in the IRIS 1_NP_ subgroup, and 46.51 U/g (IQR: 30.57–71.98 U/g) in the IRIS 1_P_ subgroup. A cut-off value of 15.80 U/g was established (Table 2). In the control group, only two dogs (11.76%) exhibited values above this threshold. Among diseased dogs, 6 out of 10 (60%) in the IRIS 1_NP_ subgroup and all dogs in IRIS stages 1_P_, 2, 3, and 4 (100%) had values exceeding this cut-off.

The uGGT/c ratio showed a strong correlation with markers of kidney disease. uGGT/c showed a positive correlation with SDMA in plasma (r = 0.591; *p* < 0.001). In urine, uGGT/c correlated positively with UPC (r = 0.716; *p* < 0.001) (Table 4).

## 4. Discussion

Chronic kidney disease (CKD) is a major complication observed in dogs with leishmaniosis, primarily associated with the deposition of immune complexes [2,4,27]. This immune complex deposition results in glomerular damage, clinically observed as persistent proteinuria, which often progresses to glomerulonephritis and/or interstitial nephritis [5]. Notably, Costa et al. (2003) [5] have demonstrated that glomerular lesions are present in all CanL-positive dogs, while interstitial nephritis occurs in 78.18% of cases, even in the absence of clinical or laboratorial abnormalities. Early detection and characterization of renal damage are crucial for initiating appropriate therapeutic interventions to prevent or delay disease progression.

The findings observed in the hematology and plasma biochemistry of the studied dogs are consistent with those expected in canine leishmaniosis [3,6,11,28,29].

Urinary gamma-glutamyl transferase (uGGT), an enzyme expressed in renal tubular cells, is a practical parameter that can be measured using most in-house analyzers. The plasma GGT, originating from the hepatobiliary system, has a molecular weight that prevents it from crossing the intact glomerular filter, thereby ensuring that urinary GGT reflects renal tubular origin exclusively [17]. An increased uGGT/c serves as an early marker for renal tubular dysfunction or damage [16,23]. Its elevation has been documented in both acute kidney injury (AKI) and CKD [30,31,32] and has been proposed as a useful diagnostic tool for detecting tubular proteinuria in canine leishmaniosis [15,16,17,33].

While published studies on the uGGT/c ratio are limited, normal values reported in healthy dogs vary between studies. The values obtained in our study (11.40 ± 7.24 U/g) align with those reported by Palacio et al. (1997) [33] (12.90 ± 4.80 U/g) and Brunker et al. (2009) [23] (13.49 ± 7.03 U/g). However, our results (median = 9.54 U/g) are lower than those reported by Perondi et al. (2019) [34] (median = 39.6 U/g) and Lippi et al. (2018) [32] (median = 31.5 U/g), although the latter two studies were conducted by the same research group.

A trend observed in healthy dogs (control group) with urinary pH < 7 showed a mean uGGT/c ratio lower than those with a urinary pH ≥ 7, consistent with Brunker et al. (2009) [23]. However, in our study, this difference was not statistically significant, likely due to the limited sample size and high individual variability. Additionally, three dogs had a urinary pH of 6, slightly below the ideal stability range for enzymatic activity [20]. This highlights the importance of considering urinary pH when interpreting uGGT activity to avoid misinterpretations.

In dogs at IRIS stage 1, uGGT/c ratio values were elevated, disregarding urinary pH. Among non-proteinuric dogs (IRIS 1_NP_), the median uGGT/c ratio was 22.15 U/g, exceeding the control group median, with 60% of cases above the established cut-off. Proteinuric dogs (IRIS 1_P_) exhibited universally higher uGGT/c ratios compared with controls, irrespective of urinary pH. Notably, two dogs with uGGT/c ratio below the cut-off exhibited urinary pH < 6.5, which may be related to enzyme inactivation.

Our findings support the hypothesis that the uGGT/c ratio is a sensitive and specific biomarker for early detection of renal tubular damage in dogs with leishmaniosis, even at initial stages without azotemia or overt proteinuria (UPC < 0.2), coinciding with previous research [16,24]. Although the uGGT/c ratio appears to be a promising early marker, it is crucial to emphasize that its definitive role in clinical practice requires further validation. The variability in results across studies, as well as the limited sample size in our own work, suggest that more research is needed before widely adopting this biomarker for routine clinical use.

A previous report has primarily focused on proteinuric dogs (UPC > 0.5), plasma creatinine and SDMA, which, while useful, have limitations in detecting early CKD [8]. In our study, pCreat showed a sensitivity of 44.2% (95% CI: 33.6–55.3%) and specificity of 100% (95% CI: 81.6–100%), while SDMA achieved a sensitivity of 51.9% (95% CI: 40.8–63%) and specificity of 100% (95% CI: 100–100%). According to the International Renal Interest Society (IRIS), a persistently elevated serum symmetric dimethylarginine (SDMA) concentration above 14 µg/dL is suggestive of early stage chronic kidney disease (CKD). In the present study, 2 dogs (7.14%) classified as IRIS stage 1 showed SDMA concentrations exceeding this threshold. However, the absence of follow-up evaluations limits the interpretation of the clinical relevance of this biomarker for detecting early stage CKD in dogs with leishmaniosis.

In contrast, the uGGT/c ratio exhibited a higher sensitivity of 90.4% (95% CI: 83.9–96.9%) compared with pCreat (44.2%; 95% CI: 33.6–55.3%) and SDMA (51.9%; 95% CI: 40.8–63.0%), with non-overlapping confidence intervals, indicating statistically significant differences. UPC also showed a relatively high sensitivity of 82.7% (95% CI: 74.3–91.1%), although its confidence interval partially overlaps with that of uGGT/c. Regarding specificity, pCreat reached 100% (95% CI: 81.6–100%), while SDMA and uGGT/c both showed 80% specificity (95% CI: 61.0–99.0%), and UPC demonstrated comparable specificity (80%; 95% CI: 61.0–99.0%). Due to the substantial overlap of confidence intervals for specificity, no statistically significant differences can be asserted among these markers in terms of their false positive rate.

In terms of overall diagnostic accuracy, the AUC for uGGT/c (0.909; 95% CI: 0.835–0.983) was the highest among all biomarkers. Although the AUCs for SDMA (0.795; 95% CI: 0.692–0.898) and UPC (0.888; 95% CI: 0.812–0.964) were also high, their confidence intervals overlapped with that of uGGT/c, preventing conclusions of statistical superiority. Only the AUC for pCreat (0.610; 95% CI: 0.483–0.738) did not overlap with that of uGGT/c, indicating significantly lower overall discriminative ability.

These findings are consistent with previous reports, such as that of de Oliveira Frazilio et al. (2018) [24], who observed that enzymuria may precede azotemia in dogs with visceral leishmaniosis. Taken together, the uGGT/c ratio shows promise as an early and sensitive biomarker for renal damage in canine leishmaniosis. Nevertheless, the overlap of confidence intervals for specificity and AUCs among SDMA and UPC underscores the need for cautious interpretation of comparative performance and supports further investigation in larger cohorts.

Palacio et al. (1997) [33] and Pardo-Marín et al. (2017) [15] reported no significant correlation between urinary gamma-glutamyl transferase/creatinine ratio (uGGT/c) and urine protein-to-creatinine ratio (UPC). However, in our cohort of dogs, we observed a highly significant positive correlation between these parameters (r = 0.716, *p* < 0.001). The precise mechanism underlying this relationship remains unclear. It has been hypothesized that excessive proteinuria may induce a competitive mechanism, resulting in increased urinary GGT levels and potentially confounding the assessment of tubular damage. The uGGT/c ratio is theoretically unaffected by such competition, unlike biomarkers that require tubular cell metabolism for detection, where protein competition is more evident [11,35,36]. GGT is not a byproduct of tubular metabolism but rather a marker of tubular cell damage and subsequent release, and, hence, severe glomerular disruption may allow plasma-derived GGT to bypass the glomerular filtration barrier. Lippi et al. (2018) [32] evaluated the utility of urinary GGT in acute kidney injury diagnosis and concluded that its discriminatory capacity between acute kidney injury and chronic kidney disease was limited, potentially due to proteinuria. Similarly, de Oliveira Frazilio et al. (2018) [24] observed a correlation between uGGT activity and the severity of proteinuria. In our study, uGGT levels reached the plateau beyond IRIS stage 2, suggesting that reduced renal excretory function may be associated with increased proteinuria and the development of tubular injury. A study conducted in Goldblatt rats with AKI due to hypertension suggested that enzymuria may decrease with the progression of tubular damage over time, as tubular enzymatic reserves become depleted and subsequent enzymatic secretion diminishes [37]. These findings emphasize the importance of assessing both UPC and enzymuria in dogs with visceral leishmaniosis, even in those with apparent normal renal excretory function [24].

In dogs with leishmaniosis, elevated uGGT/c ratios were detected in more than half of non-azotemic and non-proteinuric cases (IRIS 1_NP_: pCreat < 1.4 mg/dL; UPC = 0.12 ± 0.04) and in all non-azotemic cases with mild-to-moderate proteinuria (IRIS 1_P_: pCreat < 1.4 mg/dL; UPC = 2.12 ± 1.96). The influence of proteinuria was excluded in these cases (IRIS 1_NP_ cohort), indicating early and highly sensitive detection of tubular cell damage.

Although the IRIS classification is widely accepted as a standard for the diagnosis and staging of CKD, we acknowledge that it relies on biomarkers such as creatinine and SDMA, which are also evaluated in our study. However, we chose to use this classification as a reference because it is the most recognized and clinically relevant tool for categorizing the dogs in our study, and it provides a valid framework for dividing dogs. We also recognize that there are emerging biomarkers that could enhance early detection of renal dysfunction in dogs with leishmaniosis, and future studies may explore alternative or complementary diagnostic criteria.

The inclusion of the uGGT/c ratio in renal assessment protocols for dogs with leishmaniosis could significantly enhance the early detection and monitoring of renal injury, particularly in its initial stages. However, it is important to emphasize that, although the uGGT/c ratio seems to be a promising biomarker for early renal damage, its widespread use in clinical practice is still premature. More extensive studies are necessary to confirm its reliability and usefulness in routine clinical veterinary practice. Its ability to identify early tubular disfunction offers a broader therapeutic window, enabling interventions before irreversible kidney damage progresses [16,17]. Furthermore, its non-invasive and cost-effective nature makes it well-suited for routine veterinary clinical use. However, two factors require consideration when assessed clinically: urinary pH and the enzyme’s low stability. Clinically, urinary pH in the range of 5.5–8.5 does not appear to substantially impair the diagnostic ability of the uGGT/c ratio, though slight enzyme inactivation may occur at pH < 7. Rapid processing of samples is essential to mitigate enzymatic degradation, a limitation that can be addressed with proper sample handling.

## 5. Conclusions

The uGGT/c ratio emerges as a promising biomarker for early CKD detection in dogs with leishmaniosis, even in the absence of azotemia and with minimal or moderate proteinuria. Its potential as a novel biomarker for renal damage surpasses traditional biomarkers in sensitivity during the early stages of the disease, providing valuable insights for clinical management and improving the prognosis and quality of life for affected patients.

### Limitations and Future Considerations

A limitation of this study is the small number of non-azotemic and non-proteinuric dogs with leishmaniosis included. The sample size for both the control and diseased groups was limited by the availability of eligible dogs that met the inclusion criteria. While the small sample size may affect the statistical power and generalizability of the results, particularly in the IRIS stage 1 group, the findings still provide valuable insights into the early detection of renal damage in dogs with leishmaniosis. A larger-scale study would be necessary to improve the statistical power of the comparisons and to draw more definitive conclusions, particularly to better elucidate the influence of urinary pH on the obtained results.

Another important limitation is the absence of blinding during the clinical evaluations and laboratory analyses. The knowledge of the dogs’ group assignments (control or diseased) could have influenced the results. However, all procedures were carried out by experienced veterinarians, which minimized the potential bias. Despite this, future studies should consider using a blinded design to reduce bias and enhance the reliability of the findings.

Although strict inclusion criteria and complementary diagnostic tests were applied to exclude concurrent diseases, the presence of subclinical comorbidities cannot be completely ruled out.

Additionally, further research is warranted to evaluate the potential impact of proteinuria on uGGT/c ratio values in advanced stages of chronic kidney disease (CKD) (IRIS stages 3 and 4). While no significant interference was observed in early stages, future investigations could clarify whether massive proteinuria might lead to false-positive results due to tubular damage or allow a fraction of plasma-derived GGT to cross the glomerular filtration barrier.

Another limitation is the lack of follow-up of dogs in IRIS stage 1, which would provide more information on the progression of renal damage over time, as well as the unavailability of renal biopsies, as it is an invasive process that not all owners consent to.

## Figures and Tables

**Table 1:** Biochemical parameters in healthy dogs and in dogs with leishmaniosis categorized by IRIS staging system.

	CONTROL	IRIS 1	IRIS 1_NP_	IRIS 1_P_	IRIS 2	IRIS 3	IRIS 4
**Urea (mg/dL)**	36.19 ± 9.38	38.65 ± 19.66 ^def^	39.93 ± 17.41 ^def^	37.13 ± 21.30 ^def^	89.50 ± 69.20 *^abcef^	174.94 ± 88.65 *^abcdf^	332.64 ± 98.36 *^abcde^
**Creatinine (mg/dL)**	1.12 ± 0.11	0.97 ± 0.24 ^def^	1.02 ± 0.21 ^def^	0.92 ± 0.25 ^def^	1.57 ± 0.69 *^abcef^	3.26 ± 1.12 *^abcdf^	7.33 ± 2.70 *^abcde^
**Cholesterol (mg/dL)**	227.88 ± 54.77	207.86 ± 70.42 ^def^	180.20 ± 52.40 *^def^	223.23 ± 75.65 ^de^	274.72 ± 88.74 ^abc^	284.67 ± 84.67 ^abc^	262.69 ± 59.02 ^ab^
**Calcium (mg/dL)**	10.78 ± 0.85	11.82 ± 7.42	10.51 ± 0.58	12.52 ± 9.18	11.98 ± 6.35	10.64 ± 1.24	10.43 ± 0.74
**Phosphorus(mg/dL)**	4.50 ± 0.80	5.75 ± 8.31 ^def^	4.17 ± 1.30 ^def^	6.54 ± 10.14 ^def^	9.41 ± 14.93 *^abcef^	10.66 ± 7.15 *^abcdf^	18.41 ± 5.66 *^abcde^
**TP (g/dL)**	6.38 ± 0.53	7.27 ± 1.09 *	6.94 ± 0.90	7.46 ± 1.17 *	7.26 ± 2.16	7.45 ± 1.64	6.89 ± 1.55
**SDMA (μg/dL)**	8.60 ± 1.92	9.24 ± 3.62 ^def^	8.56 ± 3.09 ^def^	9.63 ± 3.93 ^def^	21.12 ± 5.61 *^abcef^	44.00 ± 9.55 *^abcdf^	77.00 ± 19.53 *^abcde^
**Albumin (g/dL)**	3.24 ± 0.22	2.69 ± 0.41 *^def^	2.95 ± 0.31 *^cdef^	2.55 ± 0.39 *^bed^	2.15 ± 0.50 *^abc^	2.20 ± 0.34 *^abc^	2.41 ± 0.32 *^ab^
**AG**	0.99 ± 0.25	0.64 ± 0.25 *^de^	0.81 ± 0.28 ^cdef^	0.55 ± 0.18 *^b^	0.50 ± 0.22 *^ab^	0.45 ± 0.13 *^abf^	0.58 ± 0.15 *^be^
**Globulins (g/dL)**	3.13 ± 0.39	4.58 ± 1.23 *	3.97 ± 1.08 ^c^	4.90 ± 1.21 *^b^	5.10 ± 2.14 *	5.27 ± 1.47 *	4.48 ± 1.50 *
**ALT (U/L)**	42.94 ± 14.82	40.46 ± 32.40 ^f^	37.00 ± 25.38 ^f^	42.39 ± 36.26 ^f^	34.58 ± 24.13 *^f^	66.69 ± 93.39	86.27 ± 69.06 ^abcd^
**ALKP (U/L)**	94.82 ± 42.45	134.94 ± 108.48	101.60 ± 84.40	154.55 ± 118.37	136.90 ± 151.61	129.62 ± 120.93	265.53 ± 533.78
**Sodium (mEq/L)**	150.71 ± 5.25	146.21 ± 5.87 *	147.00 ± 5.24	145.79 ± 6.29 *	146.45 ± 4.30 *	143.79 ± 5.98 *	145.87 ± 6.73 *
**Potassium (mEq/L)**	6.51 ± 9.77	4.46 ± 0.43 ^f^	4.27 ± 0.46 ^f^	4.56 ± 0.39 *^f^	4.30 ± 0.51 ^f^	4.57 ± 0.56 ^f^	5.36 ± 1.08 *^abcde^
**Chlorine (mEq/L)**	115.56 ± 4.94	110.48 ± 5.33 *	110.01 ± 5.66 *	110.73 ± 5.30 *	113.80 ± 5.56 ^f^	112.10 ± 8.50	108.66 ± 7.89 *^d^

Abbreviations: TP, total proteins; SDMA, symmetric dimethylarginine; AG, albumin/globulin ratio; ALT, alanine aminotransferase; ALKP, alkaline phosphatase. Data are expressed as mean ± standard deviation (SD). * *p* < 0.05 indicates a statistically significant difference compared with group C; ^a^ *p* < 0.05 vs. IRIS stage 1; ^b^ *p* < 0.05 vs. IRIS 1 non-proteinuric (IRIS 1_NP_); ^c^ *p* < 0.05 vs. IRIS 1 proteinuric (IRIS 1_P_); ^d^ *p* < 0.05 vs. IRIS stage 2; ^e^ *p* < 0.05 vs. IRIS stage 3; ^f^ *p* < 0.05 vs. IRIS stage 4.

**Table 2:** Cut-off points, sensitivity, and specificity of each parameter for detecting renal disease in dogs affected by leishmaniosis.

		All Dogs in Study		
	pCreat	SDMA	UPC	uGGT/c
**AUC (95% CI)**	0.610 (0.483–0738)	0.795 (0.692–0.898 **)	0.888 (0.812–0.964 ***)	0.909 (0.835–0.983 ***)
**Cut-offs**	>1.38 mg/dL	>13.5 μg/dL	>0.22	>15.8 U/g
**Sensitivity (%) (95% CI)**	44.2 (33.6–55.3)	51.9 (40.8–63.0)	82.7 (74.3–91.1)	90.4 (83.9–96.9)
**Specificity (%) (95% CI)**	100 (81.6–100)	100 (100–100)	80 (61.3–99.0)	80 (61.0–89.7)

Abbreviations: pCreat, plasmatic creatinine; SDMA, symmetric dimethylarginine; UPC, urinary proteins/creatinine ratio; uGGT/c urinary gamma-glutamyl transferase/creatinine ratio; AUC = area under the ROC curve; CI = confidence interval. ** *p* < 0.01, *** *p* < 0.001.

**Table 3:** Urinary parameters in healthy dogs and in dogs with leishmaniosis categorized by IRIS staging system.

	CONTROL	IRIS 1	IRIS 1_NP_	IRIS 1_P_	IRIS 2	IRIS 3	IRIS 4
**pH**	7.24 ± 0.75	6.63 ± 1.10	5.94 ± 0.73	7.08 ± 1.13	7.11 ± 1.11	6.55 ± 0.52	6.35 ± 1.00
**USG**	1041.5 ± 0.15	1033.2 ± 0.01 *^def^	1039.1 ± 0.01 ^def^	1030 ± 0.012 *^def^	1021.6 ± 0.09 *^abc^	1020.4 ± 0.06 *^abc^	1016.7 ± 0.07 *^abc^
**UPC**	0.14 ± 0.09	1.41 ± 1.84 *^def^	0.12 ± 0.04 ^cdef^	2.12 ± 1.96 *^bdef^	3.95 ± 2.65 *^abce^	8.06 ± 8.21 *^abcd^	8.19 ± 7.16 *^abc^
**uGGT/c (U/g)**	11.40 ± 7.24	60.15 ± 86.70 *^ef^	23.55 ± 24.03 *^cdef^	80.47 ± 102.02 *^b^	100.96 ± 116.95 *^b^	117.95 ± 103.87 *^ab^	117.64 ± 108.65 *^ab^

Abbreviations: USG, urine specific gravity; UPC, urinary proteins/creatinine ratio; uGGT/c, urinary gamma-glutamyl transferase/creatinine ratio. Data are expressed as mean ± standard deviation (SD). * *p* < 0.05 indicates a statistically significant difference compared with Group C; ^a^ *p* < 0.05 vs. IRIS Stage 1; ^b^ *p* < 0.05 vs. IRIS 1 non-proteinuric (IRIS 1_NP_); ^c^ *p* < 0.05 vs. IRIS 1 proteinuric (IRIS 1_P_); ^d^ *p* < 0.05 vs. IRIS Stage 2; ^e^ *p* < 0.05 vs. IRIS Stage 3; ^f^ *p* < 0.05 vs. IRIS Stage 4.

**Table 4:** Correlation between the biomarkers studied in the group total of dogs.

	Urea (mg/dL)	pCrea (mg/dL)	SDMA (μg/dL)	uGGT/c (U/g)	UPC
**Urea** **(mg/dL)**		0.841 ***	0.620 ***	0.294 *	0.574 ***
**pCrea** **(mg/dL)**	0.841 ***		0.611 ***	0.222 *	0.544 ***
**SDMA** **(μg/dL)**	0.620 ***	0.611 ***		0.591 ***	0.715 ***
**uGGT/c** **(U/g)**	0.294 *	0.222 *	0.591 ***		0.716 ***
**UPC**	0.574 ***	0.544 ***	0.715 ***	0.716 ***	

Abbreviations: pCrea, plasmatic creatinine; SDMA, symmetric dimethylarginine; uGGT/c, urinary gamma-glutamyl transferase/creatinine ratio; UPC, urinary proteins/creatinine ratio. * *p* < 0.05, *** *p* < 0.001.

## Data Availability

All data generated or analyzed during this study are included in this article. The datasets used and/or analyzed during the present study are available from the corresponding author upon reasonable request.

## Supplementary Materials

The following supporting information can be downloaded at https://www.mdpi.com/article/10.3390/vetsci12050436/s1, Table S1: Hematology results in healthy dogs and dogs with leishmaniosis divided by IRIS staging.

## Abbreviations

The following abbreviations are used in this manuscript:

CanL	Canine leishmaniosis
CKD	Chronic kidney disease
AKI	Acute kidney injury
ELISA	Enzyme-linked immunosorbent assay
IRIS	International Renal Interest Society
pCrea	Plasma creatinine
SDMA	Symmetric dimethylarginine
USG	Urine specific gravity
UPC	Urine protein-to-creatinine ratio
uGGT/c	Urinary gamma-glutamyl transferase/creatinine ratio
