# Supplementary material for: Urinary Gamma-Glutamil Transferase as an Early Biomarker of Renal Disease in Dogs with Leishmaniosis
Source: Vet Sci. 2025 May 2;12(5):436. doi: 10.3390/vetsci12050436 (PMC12116015; doi:10.3390/vetsci12050436)
Supplement: Supplementary file 1 [file vetsci-12-00436-s001.zip › vetsci-3573789-supplementary.pdf]

Table S1: Hematology results in healthy dogs and dogs with leishmaniosis divided by IRIS staging.

	CONTROL	IRIS 1	IRIS 1_{NP}	IRIS 1_{P}	IRIS 2	IRIS 3	IRIS 4
**RBC ($x10^6/\mu l$)**	7.15±0.91	5.80±1.31 ^{*def}	6.17±0.81 ^{*def}	5.58±1.50 ^{*def}	4.47±1.28 ^{*abc}	4.26±1.65 ^{*abc}	4.06±1.54 ^{*abc}
**Haemoglobin (g/dl)**	17.11±2.47	12.78±3.02 ^{*de}	13.90±2.36 ^{*def}	12.15±3.22 ^{*def}	9.59±2.67 ^{*abc}	9.39±3.38 ^{*abc}	9.33±3.71 ^{*bc}
**HCT (%)**	49.16±7.30	36.87±8.58 ^{*de}	39.85±6.43 ^{*def}	35.21±9.32 ^{*def}	28.02±7.42 ^{*abc}	27.37±9.12 ^{*abc}	26.56±10.93 ^{*bc}
**MCV (fl)**	68.62±3.15	63.77±3.97*	64.25±4.17*	63.50±3.95*	63.32±6.14*	66.21±9.87	64.14±4.38*
**MCHC (g/dl)**	34.81±0.85	34.53±1.40	34.83±1.16	34.36±1.52	34.15±1.40	34.09±2.19	34.41±3.26
**WBC ($x10^3/\mu l$)**	9.82±3.38	9.57±3.55 ^{f}	9.92±2.87	9.37±3.98 ^{f}	9.76±3.59 ^{f}	15.21±12.21	17.76±11.13 ^{*acd}
**Neutrophil count ($x10^3/\mu l$)**	6.11±2.48	6.50±3.03 ^{f}	6.20±2.30 ^{f}	6.66±3.42 ^{f}	7.08±2.88 ^{f}	9.87±7.85	14.89±9.78 ^{*abcd}
**Eosinophil count ($x10^3/\mu l$)**	0.47±0.31	0.31±0.28 ^{*f}	0.33±0.29 ^{f}	0.29±0.28 ^{*f}	0.28±0.21 ^{*f}	0.22±0.30*	0.09±0.08 ^{*abcd}
**Basophil count ($x10^3/\mu l$)**	0.03±0.03	0.06±0.03	0.05±0.04	0.03±0.03 ^{f}	0.05±0.53	0.05±0.09 ^{f}	0.06±0.05 ^{ce}
**Lymphocyte count ($x10^3/\mu l$)**	2.70±2.31	2.02±1.10	2.81±1.26 ^{cdef}	1.58±0.71 ^{b}	1.61±0.83 ^{b}	1.57±1.10 ^{b}	1.45±0.78 ^{*b}
**Monocyte count ($x10^3/\mu l$)**	0.52±0.36	0.71±0.45 ^{f}	0.54±0.32 ^{ef}	0.80±0.50	0.74±0.41	1.55±1.53 ^{*b}	1.27±0.98 ^{*ab}
**PTL ($x10^3/\mu l$)**	222.29±84.04	229.82±142.85	168.70±76.77	263.78±160.84 ^{e}	199.21±122.16	172.00±157.33 ^{*c}	195.94±114.49

Abbreviations: RBC, red blood cell; HCT, haematocrit; MVC, medium corpuscular volume; MCHC, mean corpuscular haemoglobin concentration; WBC, white blood cell; PTL, platelet. Values are presented as mean ± SD.

** P < 0.05 differ statistically with Group C*

^{a} P < 0.05 differ statistically with IRIS 1

^{b} P < 0.05 differ statistically with IRIS 1_{NP}

^{c} P < 0.05 differ statistically with IRIS 1_{P}

^{d} P< 0.05 differ statistically with IRIS 2

^{e} P< 0.05 differ statistically with IRIS 3

^{f} P< 0.05 differ statistically with IRIS 4
